# A snapshot into the transcriptomic landscape of apoptosis and ferroptosis in cancer

**DOI:** 10.1038/s41419-024-06766-8

**Published:** 2024-05-29

**Authors:** Yaron Vinik, Sima Lev

**Affiliations:** Molecular Cell Biology Department, Weizmann Institute of Science, Rehovot, Israel

**Keywords:** Cell death, Biomarkers

## Abstract

Apoptosis and ferroptosis are two regulated cell death (RCD) pathways implicated in different human diseases and considered as promising strategies to eliminate cancer cells. These two pathways are characterized by distinct morphological and biochemical properties, induce cell death through different mechanisms, but share common regulators in different cancer types. Although apoptosis and ferroptosis have been extensively studied over the last few years, their transcriptomic responses have not yet been systematically compared due to remarkable variability in the transcriptomic data. Here we provide a brief snapshot of the transcriptomic landscapes of the apoptosis and ferroptosis responses in cancer, discuss their divergent and convergent properties, and implications to cancer therapy.

The importance of apoptosis in cancer therapy has been demonstrated by numerous pre-clinical and clinical studies, and by the Food and Drug Administration approval of several small molecule inhibitors against anti-apoptotic proteins of the BCL2 family as therapeutic targets for certain cancer types [1]. Apoptotic death is also induced by chemotherapy, radiotherapy, immunotherapy, and many targeted therapies. However, the emerging resistance to specific pro-apoptotic drugs or other apoptosis-associated therapeutic modalities, highlights the need to target alternative regulated cell death pathways. In recent years, ferroptosis has been proposed as a promising strategy for cancer therapy, augmenting immunotherapy responses and overcoming resistance to various anti-cancer drugs [2]. Ferroptosis might be especially beneficial for cancers that are more susceptible to this death pathway [3], such as the highly aggressive triple-negative breast cancer (TNBC) [4, 5].

Extensive studies on apoptosis, since it was first described approximately 52 years ago [6], indicate that the pathway is characterized by unique morphological properties, including chromatin condensation, DNA fragmentation, cell rounding and shrinkage, membrane blebbing and apoptotic body formation [7]. Further biochemical studies have revealed that apoptosis is mainly mediated by the activation of aspartate-specific cysteine proteases of the caspase family. Ferroptosis, on the other hand, which was described approximately 12 years ago [8], is characterized by the loss of membrane integrity and changes in the mitochondrial ultrastructure. Ferroptosis is mediated by iron-dependent lipid peroxidation of poly unsaturated fatty acids and is initiated by oxidative perturbations, which are primarily protected by the antioxidant cellular machinery and by glutathione peroxidase 4 (GPX4) [9, 10].

Despite the differences between the two death modules, increasing evidence suggest that ferroptosis and apoptosis can be triggered by similar stress signals such as oxidative stress and endoplasmic reticulum (ER) stress. The pathways also share common regulators, such as p53, and may occur in the same dying cells either sequentially or simultaneously and can even cooperate to induce cell death [11]. The tumor suppressor p53, for example, which is activated in response to various stress signals, directly regulates the transcription of the pro-apoptotic genes Puma/Bbc3 and Noxa/Pmaip [12], and concomitantly can suppress the expression of the anti-ferroptosis protein SLC7A11, a subunit of the cysteine–glutamate antiporter, which plays key role in cystine uptake and GSH metabolism. P53 also regulates the expression of SAT1 (spermidine/spermine acetyltransferase), leading to upregulation of the pro-ferroptotic protein ALOX15 (arachidonic acid lipoxygenase 15) [13]. Although the effects of p53 could be context dependent, p53 was proposed to play central role in the crosstalk between ferroptosis and apoptosis. The crosstalk between the two death modules is also reflected in the function of different intracellular organelles, such as the lysosome, the mitochondria, and the ER [14].

While the biochemical and signaling crosstalk between ferroptosis and apoptosis has been described and is continuously studied in various cancer cells, little is known about the crosstalk between the transcriptomic response of ferroptosis and apoptosis. The major challenge in comparing the transcriptomic landscapes of the two death modules is associated with the remarkable variability of the transcriptomic data, possibly due to (i) the diverse apoptosis and ferroptosis inducers and their distinct modes of action, (ii) the various biological models and experimental settings, and (iii) the different kinetics of the transcriptomic response.

To overcome these challenges, we collected a relatively large number of transcriptomic datasets from different cancer models (cells and tumors) in response to ferroptosis inducers (FINs) and apoptosis inducers (AINs), 49 datasets in total, 23 for FINs and 26 for AINs (Fig. 1A). Our analysis of the differentially expressed genes (DEGs) in the various datasets revealed remarkable variability within the transcriptomic landscapes of FINs and AINs, which appeared to be more dependent on the biological model rather than the identity of the inducer (Fig. 1B). The canonical FIN Erastin, for example (Fig. 1B), upregulated ~10-fold more genes in hepatocellular carcinoma [15] than in fibrosarcoma [16].

**Fig. 1:**
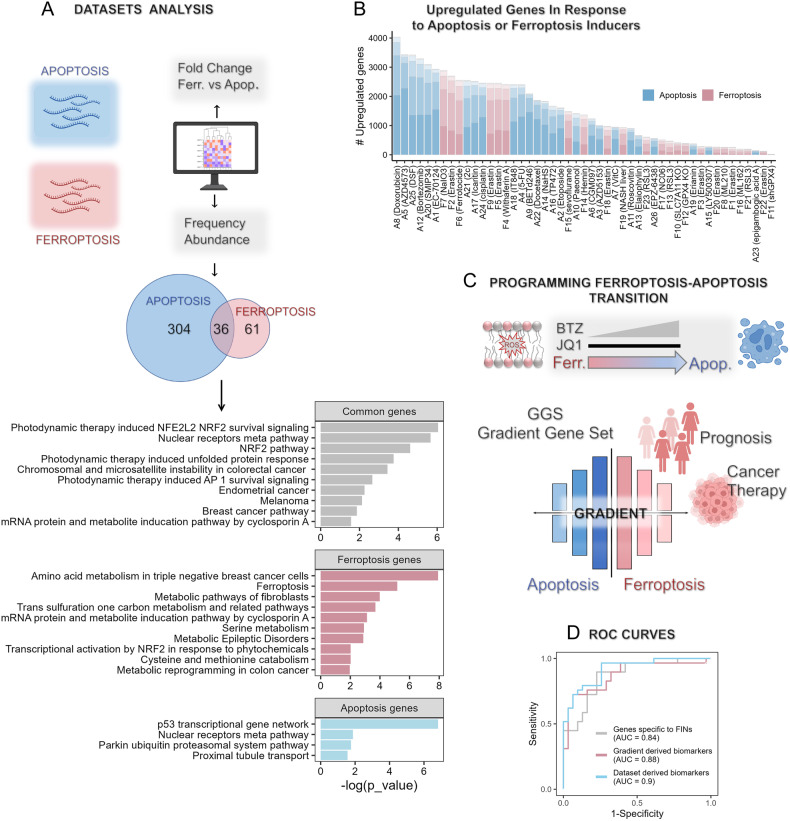
The transcriptomic landscapes of ferroptosis and apoptosis. **A** Dataset analysis. 49 datasets, 23 in response to FINs and 26 in response to AINs, were normalized and analyzed for DEGs of inducers versus controls. Two methods were used to analyze the ferroptosis versus apoptosis transcriptomic response: (1) comparing the fold-changes in each gene in the FINs versus the AINs; (2) quantifying the percentage of datasets in which each gene was significantly upregulated (‘Frequency Abundance’). The Venn diagram depicts the number of genes significantly upregulated in at least 40% of the FIN datasets or the AIN datasets, or in both (common genes). Shown below the Venn diagram is the pathway enrichment analysis for these genes, performed by gProfiler2 in R. **B** Variability in the transcriptomic responses. The variability is demonstrated by the variable number of significantly upregulated genes in each dataset; the 23 ferroptosis (pink) and 26 apoptosis (blue). The hue of the color reflects the frequency of significant genes among all datasets. The lightest color represents upregulated genes in at least 50% of the FINs or AINs datasets, and the darkest color represents genes upregulated in less than 10% of the datasets. **C** Programming a ferroptosis-to-apoptosis transition by drug combinations. A drugs combination of JQ1 (targeting BRD4) and bortezomib (BTZ, a proteasome inhibitor) was used to identify a gradual ferroptosis-to-apoptosis transcriptomic shift, by slightly modifying the concentration of BTZ while maintaining the JQ1 dose low and constant. The resulting gene signature, the “Gradient Gene Set (GGS),” and/or its specific subsets were used to classify ferroptosis and apoptosis datasets, shown to have a prognostic value in breast cancer patients, and consists of ferroptosis repressors, which might be used for breast cancer therapy. **D** Classification accuracy of gene signatures. The described signatures identified by the three methods (two are shown in (**A**) and one in (**C**)), labeled as “Genes specific to FINs” (those that are significantly upregulated in at least 40% of the datasets, AUC = 0.84, 95% CI 0.76–0.97), “Dataset-derived genes” (those genes with the highest fold change between the FIN and AIN datasets, AUC = 0.9, 95% CI 0.81–0.98), and the “Gradient-derived genes” (optimized subset of 15 genes of the GGS, AUC = 0.88, 95% CI 0.75–0.97) were used to classify the 49 FIN and AIN datasets. All AUCs of the classification between the FINs and AINs are significant.

Next, we looked for death module-specific genes, which may regulate unique processes associated with the death response compared to common genes shared by the two pathways. The common upregulated genes may represent common stress response genes, compensating genes, or genes that play roles in the crosstalk between the death pathways.

Two approaches were applied to identify death module-specific versus common genes (Fig. 1A). The first approach relied on the frequency of the datasets (at least 40%) in which the upregulated genes were significant among the 23 FIN or the 26 AIN datasets, while the second approach relied on the fold-changes in gene expression between the ferroptosis and apoptosis datasets. These two approaches identified death module-specific genes as well as death modules common genes (Fig. 1A), some of them were concomitantly identified by both approaches. Pathway enrichment analysis of the common upregulated genes highlighted the NRF2 targets, consistent with the involvement of oxidative stress in both death modules. This analysis implies that some of the common upregulated genes are involved in the crosstalk between ferroptosis and apoptosis. Amino acid metabolism and the transsulfuration pathways were enriched in response to FINs, while genes associated with the ubiquitin proteasomal response and with p53, were enriched in response to AINs (Fig. 1A). These findings are consistent with the role of these specific pathways in each respective death module [17].

Identifying death-module discrete genes is extremely important, as these genes can be used as reliable transcriptomic classifiers to distinguish between the two death modules in response to cancer treatment or in drug repurposing. Indeed, the ferroptosis-selective genes identified by the two described approaches could classify the 49 ferroptosis and apoptosis transcriptomic responses with high and significant accuracy (area under the curve (AUC) = 0.84 and 0.9) (Fig. 1D). Nevertheless, due to the high variability in the transcriptomic responses to FINs, ferroptotic death should be defined based on the transcriptomic response of a subset of biomarkers instead of individual genes (by quantitative polymerase chain reaction, for example). This is particularly important when considering the different magnitude of the fold-change and kinetics of gene expression even in response to the same inducer.

While these classifications rely on the largely variable 49 datasets (Fig. 1B), in our recent study published in *Advanced Science* [18], we used an innovative, unbiased approach to program a continuous transcriptomic landscape between ferroptosis and apoptosis in basal-like breast cancer cells (Fig. 1C). This transitional landscape was established by titrating a synthetic lethal drugs combination of JQ1 and bortezomib, targeting BRD4 and the proteasome, respectively. This drug combination induced ferroptosis at very low doses in TNBC cell lines across different molecular subtypes [19], while a slight increase in bortezomib concentration shifted the death to apoptosis [18]. Programming the ferroptosis-to-apoptosis transition by closely related ferroptosis and apoptosis inducers (i.e., the same two drugs but with a slight different dose of one of them), in the same experimental model and time points, allowed us to capture the transcriptomic landscape of the two death modules with minimal variability (Fig. 1C), since the variability of the applied inducers, of the biological model, and of the time points of the different datasets (Fig. 1B) was substantially reduced.

Indeed, we found a remarkable overlap in the transcriptomic response (80%) of the two death models induced by the drug combinations. These close transcriptomic responses were used to organize the DEGs in a gradual manner and to identify a unique “Gradient Gene Set” (GGS) (Fig. 1C). The GGS of 306 genes as well as its smaller subset of 15 genes (Fig. 1D) could also classify ferroptosis and apoptosis with high accuracy (AUC = 0.88, Fig. 1D).

Further optimization of the GGS eventually led to the identification of 24 ferroptosis biomarkers, which were robustly validated in cultured cells and a mice model of breast cancer [18]. The identified ferroptosis versus apoptosis selective biomarkers could be extremely valuable as a group of biomarkers for monitoring ferroptotic death in different pathological conditions or in response to cancer treatment. Indeed, a subset of the GGS representing upregulated genes in response to ferroptosis induction was highly correlated with poor prognosis in breast cancer patients and could predict cancer aggressiveness and response to chemotherapy. In addition, it includes several ferroptosis repressors that might be promising candidates for breast cancer therapy, of which one representative, PDAP1 (PDGFA-associated protein 1), was validated in a xenograft tumor model [18].

Collectively, the transcriptomic response of regulated-cell-death pathways, specifically of apoptosis and ferroptosis, and the identification of death-specific biomarkers could be used to monitor death responses to anti-cancer drugs, to develop strategies for overcoming drug resistance, and to identify potential novel, effective targets for cancer therapy.

## Concluding remarks

- The transcriptomic responses of ferroptotic and apoptotic deaths are highly variable.
- The high variability is influenced by the diversity in the ferroptosis and apoptosis inducers, by the different experimental models and settings, and by the different kinetics of the responses.
- The model system has a strong influence on the transcriptomic response.
- The death module-specific responses can be identified through comparison of multiple transcriptomic datasets and can highlight death-specific regulatory pathways.
- Genes common to both death modules may represent common stress response genes, co-regulators, and crosstalk pathways.
- The programming of a continuous landscape between death modules can be applied to identify death transcriptomic classifiers and to monitor specific death responses.
- The death module-specific upregulated gene set includes a few death repressors, whose targeting might be used for cancer therapy.

## References

[1] Carneiro BA, El-Deiry WS (2020). Targeting apoptosis in cancer therapy. Nat Rev Clin Oncol.

[2] Lei G, Zhuang L, Gan B (2024). The roles of ferroptosis in cancer: tumor suppression, tumor microenvironment, and therapeutic interventions. Cancer Cell.

[3] Zhang C, Liu X, Jin S, Chen Y, Guo R (2022). Ferroptosis in cancer therapy: a novel approach to reversing drug resistance. Mol Cancer.

[4] Lev S (2020). Targeted therapy and drug resistance in triple-negative breast cancer: the EGFR axis. Biochem Soc Trans.

[5] Li J, He D, Li S, Xiao J, Zhu Z (2023). Ferroptosis: the emerging player in remodeling triple-negative breast cancer. Front Immunol.

[6] Kerr JF, Wyllie AH, Currie AR (1972). Apoptosis: a basic biological phenomenon with wide-ranging implications in tissue kinetics. Br J Cancer.

[7] Lamb HM (2020). Double agents of cell death: novel emerging functions of apoptotic regulators. FEBS J.

[8] Dixon SJ, Lemberg KM, Lamprecht MR, Skouta R, Zaitsev EM, Gleason CE (2012). Ferroptosis: an iron-dependent form of nonapoptotic cell death. Cell.

[9] Tang D, Kang R, Berghe TV, Vandenabeele P, Kroemer G (2019). The molecular machinery of regulated cell death. Cell Res.

[10] Stockwell BR (2022). Ferroptosis turns 10: emerging mechanisms, physiological functions, and therapeutic applications. Cell.

[11] Li Z, Chen L, Chen C, Zhou Y, Hu D, Yang J (2020). Targeting ferroptosis in breast cancer. Biomark Res.

[12] Aubrey BJ, Kelly GL, Janic A, Herold MJ, Strasser A (2018). How does p53 induce apoptosis and how does this relate to p53-mediated tumour suppression?. Cell Death Differ.

[13] Zhan J, Wang J, Liang Y, Zeng X, Li E, Wang H (2023). P53 together with ferroptosis: a promising strategy leaving cancer cells without escape. Acta Biochim Biophys Sin.

[14] Wu P, Zhang X, Duan D, Zhao L (2023). Organelle-specific mechanisms in crosstalk between apoptosis and ferroptosis. Oxid Med Cell Longev.

[15] Zhang X, Du L, Qiao Y, Zhang X, Zheng W, Wu Q (2019). Ferroptosis is governed by differential regulation of transcription in liver cancer. Redox Biol.

[16] Dixon SJ, Patel DN, Welsch M, Skouta R, Lee ED, Hayano M (2014). Pharmacological inhibition of cystine-glutamate exchange induces endoplasmic reticulum stress and ferroptosis. Elife.

[17] Stockwell BR, Friedmann Angeli JP, Bayir H, Bush AI, Conrad M, Dixon SJ (2017). Ferroptosis: a regulated cell death nexus linking metabolism, redox biology, and disease. Cell.

[18] Vinik Y, Maimon A, Dubey V, Raj H, Abramovitch I, Malitsky S (2024). Programming a ferroptosis-to-apoptosis transition landscape revealed ferroptosis biomarkers and repressors for cancer therapy. Adv Sci.

[19] Verma N, Vinik Y, Saroha A, Nair NU, Ruppin E, Mills G (2020). Synthetic lethal combination targeting BET uncovered intrinsic susceptibility of TNBC to ferroptosis. Sci Adv.

